# Aerotolerancy of *Campylobacter* spp.: A Comprehensive Review

**DOI:** 10.3390/pathogens13100842

**Published:** 2024-09-28

**Authors:** Elise Delaporte, Anand B. Karki, Mohamed K. Fakhr

**Affiliations:** 1 Department of Biological Science, The University of Tulsa, Tulsa, OK 74104, USA; elisead@utulsa.edu; 2 Department of Biological Sciences, Sam Houston State University, Huntsville, TX 77341, USA

**Keywords:** *Campylobacter*, aerotolerance, growth media, oxidative stress, virulence

## Abstract

*Campylobacter* spp. constitute a group of microaerophilic bacteria that includes strains that are aerotolerant and capable of surviving in aerobic conditions. Recent studies have shown that aerotolerant strains are highly prevalent in meats, animals, and clinical settings. Changes in growth media and other environmental conditions can affect the aerotolerance of *Campylobacter* strains and must be considered when studying their aerotolerance in vitro. Polymicrobial interactions and biofilms also play a significant role in the ability of *Campylobacter* to survive oxygen exposure. Continuous subculturing may foster aerotolerance, and studies have demonstrated a positive correlation between aerotolerance and virulence and between aerotolerance and the ability to survive stressful environmental conditions. Various mechanisms and genetic origins for aerotolerance have been proposed; however, most of the potential genes involved in aerotolerance require further investigation, and many candidate genes remain unidentified. Research is also needed to investigate if there are any clinical implications for *Campylobacter* aerotolerance. Understanding the aerotolerance of *Campylobacter* remains an important target for further research, and it will be an important step towards identifying potential targets for intervention against this clinically important food-borne pathogen.

## 1. Introduction

*Campylobacter* spp. are the cause of campylobacteriosis, which is considered the most common source of bacterial gastroenteritis worldwide [1,2,3,4]. Most cases of campylobacteriosis are mild and self-limiting, but infection may result in death for young children, the immunosuppressed, and the elderly [5,6]. Occasionally, fatalities have been observed in seemingly healthy adults, but these are rare [5]. Severe complications such as Guillain-Barré syndrome, reactive arthritis, and irritable bowel syndrome can occur as a consequence of campylobacteriosis [7]. Common food sources that may be contaminated with *Campylobacter* include undercooked meats, raw dairy products, and produce, and most cases in the United States occur due to contact with raw or undercooked poultry [8,9]. Around 80–85% of campylobacteriosis cases are caused by *C*. *jejuni*, but *C*. *coli*, *C*. *fetus*, and other *Campylobacter* spp. are also known to cause illness in humans [10].

*Campylobacter* spp. are Gram-negative, microaerophilic bacteria with an optimal growth temperature ranging from 37 to 42 °C [8,11]. Microaerophilic bacteria prefer oxygen concentrations of 2–10% and generally do not grow well at higher O_2_ levels [12]. Due to their microaerophilic nature, we would expect normal atmospheric oxygen concentrations to be fatal for *Campylobacter*; however, despite their specialized growth requirements, *Campylobacter* spp. are prevalent during meat processing [13]. Therefore, we can assume that *Campylobacter* is able to survive and potentially multiply in oxygen-rich environments. As we search for ways to reduce the contamination of meat products, a better understanding of aerotolerance in *Campylobacter* is critical.

Recent studies on *Campylobacter* aerotolerance have focused on the following areas: (1) the prevalence of aerotolerant *Campylobacter* strains in meat production; (2) the effects of specific growth media, supplements, or environmental factors on aerotolerance; (3) the correlation between aerotolerance and other survival traits such as virulence; and (4) the mechanistic basis of aerotolerance, including the genes and proteins responsible for aerotolerance. Summarizing the existing literature is crucial for identifying gaps in knowledge and guiding future research directions. It also allows for the identification of key insights and patterns that may otherwise be overlooked. The aim of this review is to synthesize past research on the aerotolerance of *Campylobacter* and provide recommendations for future investigations and research strategies.

## 2. Prevalence of Aerotolerant *Campylobacter* Strains in Meat Production

Recently, a high prevalence of aerotolerance has been identified in *Campylobacter* isolated from meat, animal, and human sources [14,15,16,17,18,19,20,21,22]. These studies highlight the importance of studying aerotolerance to better reduce the incidence of *Campylobacter* contamination in food. For the purposes of this review, an “aerotolerant” strain is defined as a strain capable of surviving at least 12 h of exposure to aerobic conditions, and a “hyper-aerotolerant” strain can survive 24 h of exposure to aerobic conditions [17]. These parameters were established based on the prevalence of aerotolerant and hyper-aerotolerant strains described in the literature [14,15,16,17,18,19,20,21,22].

The prevalence of aerotolerant and hyper-aerotolerant strains of *C*. *jejuni* varies widely among studies, possibly due to the source of the isolate or country of origin [14,15,16,17,18,19,21,22]. In studies that focused on the prevalence of aerotolerance in *C*. *jejuni*, the occurrence of aerotolerant strains ranged from 6.5 to 98.6%, and these were isolated from chicken, duck, turkey, and pork meat, beef and chicken liver, chicken gizzards, chicken carcasses, chicken feces, a broiler processing plant, human patients, broilers, and cattle [14,15,16,17,18,21,22]. Meanwhile, the occurrence of hyper-aerotolerant *C*. *jejuni* strains ranged from 0 to 65.5% [14,15,16,17,18,19,21,22]. In studies of *C*. *coli* prevalence, the incidence of aerotolerant and hyper-aerotolerant strains ranged from 41.9 to 89.3% and 13.7 to 52%, respectively, and were isolated from chicken, pork, turkey, and duck meat, duck and broiler carcasses, chicken and beef liver, chicken gizzards, dairy products, human patients, and swine [19,20,21,23]. Contradictory studies exist in terms of whether aerotolerance in *C*. *jejuni* or *C*. *coli* is more prevalent [19,21], which could be due to the isolation of strains from different countries and sources. Although the aerotolerance of *Campylobacter lari* is not well-studied, at least one hyper-aerotolerant *C*. *lari* strain has been described [24]. The high variance in the occurrence of aerotolerant or hyper-aerotolerant strains could be due to location, the source, or other factors, such as the methods used for isolating the strains.

As discussed later in this review, the media used for culturing *Campylobacter* is an important consideration when comparing data from different studies because it can affect aerotolerance [25,26,27,28,29,30,31,32,33,34]. Researchers studying prevalence often use Mueller–Hinton agar (MHA) for isolating strains and enumeration and Mueller–Hinton broth (MHB) for evaluating aerotolerance, but there are exceptions [14,15,16,17,18,19,20,21,22,23,24]. To elaborate further, one research group used MHA supplemented with 5% horse blood for isolating cultures [21]. In another study, both MHA and MHB were used, but bacteria were enumerated by plating on Preston *Campylobacter* selective agar [19], which is a blood-containing medium [35]. Apparently, adding blood to the media increases *Campylobacter* aerotolerance [30,34,36], which could impact the enumeration of bacterial strains. In some reports, the media used for bacterial enumeration was not explicitly stated [15,16,17]. Another report describes isolating strains on Oxoid *Campylobacter* selective agar, conducting aerotolerance testing with brain heart infusion broth, and enumerating colonies on *Campylobacter* selective agar [18], It is important to note that *Campylobacter* selective agar contains blood, ferrous sulfate, sodium pyruvate, and sodium metabisulfite [1,37]; these ingredients are known to enhance *Campylobacter* growth and increase its survival during oxygen exposure, which could impact enumeration [26,30,34,36,38], Furthermore, supplementation of media with juice taken from other organs, such as the liver, can increase aerotolerance [39]; thus, it remains possible that the organs in brain heart infusion broth could improve aerotolerance and skew the results. One report deviates from convention by conducting aerotolerance testing in Oxoid™ Nutrient Broth No. 2 (Thermo Fisher Scientific) supplemented with 1.5% Bacto agar from BD Biosciences [14]. According to Thermo Fisher, Oxoid™ Nutrient Broth No. 2 contains 10.0 g/L meat extract. In contrast, the Mueller–Hinton Broth manufactured by Thermo Fisher contains 2.0 g/L of beef extract, and the latter was shown to help *Campylobacter* growth in aerobic conditions [40], which could impact the results. Furthermore, the same study reported an extremely high incidence of aerotolerant *C*. *jejuni*, with 98.6% of the strains (69/70) exhibiting aerotolerance [14]; however, another study also reported a high prevalence (over 90%) of aerotolerant strains [16]. Overall, the relative consistency of the growth media used for aerotolerance testing in recent studies is encouraging, but further standardization of growth conditions would be beneficial.

## 3. Effects of Environmental Conditions on Aerotolerance

### 3.1. Growth Media and Supplementation

As noted above, multiple studies have described the effect of growth media and supplements on the aerotolerance of *Campylobacter*, which indicates that incubation conditions should be considered when evaluating aerotolerance. One supplementation strategy involves the addition of iron-containing compounds to the growth media. For example, the addition of iron to the growth media in the form of blood or as a ferrous salt enhanced aerotolerance in *Campylobacter* [25,30,34,36]. Interestingly, norepinephrine is known to enhance the growth of *C*. *jejuni* by stimulating iron uptake [33], and *Campylobacter* aerotolerance increased when norepinephrine was present [25]. Furthermore, iron supplementation stimulated biofilm formation in *C*. *jejuni* [41]; since biofilms contribute to aerotolerance [42,43,44,45], it remains possible that iron increases aerotolerance by stimulating biofilm formation. It is noteworthy that the expression of certain genes involved in the oxidative stress response of *C*. *jejuni* is also impacted by iron [46,47,48]. In addition to acting as an iron source, blood also contains antioxidant enzymes such as superoxide dismutase (SOD) [49], and supplementation of growth media with oxidative stress enzymes such as SOD and catalase increased aerotolerance in *C*. *jejuni* [27]. The ability of catalase to enhance aerotolerance has been confirmed [29]; however, two subsequent studies showed that the addition of SOD did not significantly increase aerotolerance [29,32]. Supplementation with Oxyrase increased *C*. *jejuni* growth in normal atmospheric conditions, especially in the presence of blood [34,50]. Oxyrase, which has been used to promote the growth of anaerobic bacteria, is generated from membrane fragments of *E*. *coli* and can remove dissolved oxygen from the growth media [51].

In addition to blood, other animal-derived products are beneficial for aerobic growth, such as supplementation with 5% or 7.5% beef extract [40]. Juices from chicken or beef liver also enhanced aerotolerance in *C*. *jejuni* and *C*. *coli*, and the addition of 10% chicken or beef liver juice increased aerotolerance more than supplementation with blood or meat juice from other tissues [39]. In addition to blood, liver juice apparently contains other components that heighten aerotolerance [39]. Collectively, these studies suggest that meat constituents help *Campylobacter* survive oxygen exposure during meat processing.

In addition to iron and meat, other compounds are known to increase aerotolerance. For example, pyruvate is potentially beneficial to aerotolerance [32,52], and its addition decreased catalase activity in *C*. *jejuni* [52]. C3-monocarboxylates like pyruvate and C4-dicarboxylates such as fumarate and sodium bicarbonate were beneficial for aerobic growth [53], and a later study confirmed that supplementation of media with fumarate-pyruvate enhanced the aerobic growth of *Campylobacter* [40]. Supplementation with 0.01% bisulfite increased *Campylobacter* survival in aerobic conditions [28]. Since bisulfite enhanced *C*. *jejuni* growth in an oxygen-free environment, the authors speculate that bisulfite is not simply an oxygen scavenger but benefits the bacteria via an unknown function [28]. In Brucella agar, bisulfite deteriorated over time in both dehydrated and hydrated media, and its degradation decreased *Campylobacter* aerotolerance [31]. Furthermore, the addition of 0.01% bisulfite to the medium reversed deterioration [31], which suggests that the age of the media could impact the outcome of aerotolerance testing. Combining an iron salt with pyruvate and bisulfite was beneficial to aerotolerance, and multiple reports demonstrated that a combination of ferrous sulfate, sodium bisulfite, and sodium pyruvate (FBP) or supplementation with charcoal, ferrous sulfate, and sodium pyruvate increased *Campylobacter* aerotolerance [26,27,30,34,36,38]. Adding iron sources such as 5% laked horse blood along with FBP increased aerotolerance to even higher levels [34].

*C*. *jejuni* can transition from its normal morphology to a coccoid form in the presence of H_2_O_2_ or O_2_, and supplementation with FBP reduced conversion to the coccoid morphology in the presence of O_2_ [30,38,54,55,56]. The addition of SOD or blood also reduced the conversion to the coccoid morphology [30,56], and reducing the concentration of serine increased oxygen sensitivity in *C*. *jejuni* [57]. The effect of multiple supplements on *C*. *jejuni*, *C*. *coli*, and *C*. *fetus* was examined, and allopurionol, azelaic acid, caffeine, cimetidine, and TEMPOL enhanced aerotolerance, whereas carnosine, dimethyl thiourea, and spermidine had little effect on aerotolerance [32]. Another study reported that supplemental dithionite and histidine increased aerotolerance in *C*. *jejuni* in response to light and air, whereas cysteamine, α-tocopherol, and butylated hydroxytoluene were not beneficial [29].

The effects of different sources of complex media on aerotolerance have been examined [32]. Two different brands of tryptic soy agar (TSA) were compared, and the growth of *Campylobacter* in oxygenated conditions varied with the source. One brand of TSA permitted *Campylobacter* growth at both 15% and 21% oxygen, whereas the other brand did not [32]. Furthermore, two sources of ingredients were used to create a bisulfite-free formulation of Brucella agar, and only one brand allowed growth at O_2_ concentrations of 15% and 21% [32]. Changing the source of tryptone used in media formulation also altered the O_2_ tolerance of *Campylobacter* [32]. The study concluded that the use of different brands of complex media can result in large variations in aerotolerance, and it was concluded that a chemically defined medium may be more desirable for consistency [32]. These studies illustrate the importance of standardizing the media formulation used in aerotolerance testing to produce accurate results that can be compared with other studies.

### 3.2. Effects of Polymicrobial Interactions and Biofilms

Beyond the effects of growth media on aerotolerance, the presence of other microorganisms has also been shown to increase the aerotolerance of *C*. *jejuni*. For example, co-cultivation of *C*. *jejuni* with *Pseudomonas* spp., *Staphylococcus aureus*, or *Salmonella* allowed *Campylobacter* to survive aerobic conditions for longer periods [58,59,60]. Interestingly, co-cultivation with *Bacillus subtilis* had a protective effect on *C*. *jejuni* survival under aerobic conditions but inhibited its growth in microaerobic conditions [61]. Even supplementation with cell fractions of other bacterial strains may improve aerotolerance; for example, supplementation with membrane fragments from *Pseudomonas aeruginosa* and *Escherichia coli* increased the growth of *C*. *jejuni* in atmospheric oxygen [62]. Similarly, cell-free media obtained from *S*. *aureus* increased *Campylobacter* survival during oxygen exposure [60]. Culturing with eukaryotic organisms can also be beneficial; for example, multiple studies have shown that co-cultivation of *Campylobacter* with amoebas is beneficial in aerobic conditions and does not require the addition of blood [63,64]. Co-cultivation of *C*. *jejuni* with amoebas increased the extracellular aerobic survival of *C*. *jejuni* even when the two organisms were not in direct contact; this was due to the reduction of dissolved oxygen in the media [64]. Even the addition of bacteriophages may enhance aerotolerance; for example, exposing *C*. *jejuni* to bacteriophages during limited nutrient intake caused an increase in aerotolerance; however, bacterial motility and colonization of host chickens were impaired as a result [65]. Understanding the impact of polymicrobial interactions on aerotolerance is crucial as implementing control measures against other organisms that enhance *Campylobacter*’s aerotolerance could have the desirable side effect of reducing *Campylobacter*’s survival, as well. However, it is essential to evaluate the influence of various microorganisms on *Campylobacter* as certain microorganisms, such as some probiotics, may also have an inhibitory effect on *Campylobacter*’s survival [66].

Biofilm formation was previously found to enhance the survival of *C*. *jejuni* during oxidative stress [44,67,68]. Multiple studies have shown that *Campylobacter* displays enhanced biofilm formation when grown in O_2_-enriched conditions [42,43,45,55,69]; furthermore, hyper-aerotolerant strains of *C*. *jejuni* have an enhanced ability to form biofilms in aerobic conditions [19]. *Campylobacter* cells cultured in O_2_-rich environments have greater adhesive properties that could potentially contribute to biofilm formation [70]. Interestingly, supplementation with formic acid decreased both aerotolerance and biofilm formation in *C*. *jejuni* grown in aerobic conditions, thus illustrating a relationship between aerotolerance and biofilm formation [71]. Moreover, polymicrobial biofilms were more helpful to *C*. *jejuni* survival in aerobic conditions than monocultural biofilms [72]. Polymicrobial interactions with other bacteria enhanced biofilm formation in *C*. *jejuni* [55,60,69,72,73,74,75,76], indicating that biofilm formation is beneficial to aerotolerance. This research has significant implications for food safety practices, as identifying strategies to disrupt biofilm formation during food processing may reduce *Camplyobacter*’s aerotolerance, thereby inhibiting its ability to survive meat processing.

### 3.3. Influence of Other Environmental Factors on Aerotolerance

Temperature is another important factor that should be considered when assessing aerotolerance. For example, the aerotolerance of *C*. *jejuni* increased when cells were cultivated at 42 °C as compared to 37 °C [31]. Other researchers reported that *C*. *jejuni* is more sensitive to oxidative stress at 42 °C and 37 °C vs. lower temperatures [11,77], and survival in 21% O_2_ was greater at 4 °C than at 25 °C [28]. Temperature clearly impacts aerotolerance and should be kept consistent when comparing different strains. Additionally, the increase in aerotolerance observed at low temperatures suggests that the refrigeration of poultry during meat processing may inadvertently be enhancing *Campylobacter*’s ability to survive oxygen exposure.

Other environmental factors also impact aerotolerance. A very high concentration of CO_2_ (>97%) significantly reduced colonization of poultry by a hyper-aerotolerant *C*. *jejuni* strain and is a promising approach for inhibiting colonization during processing [78]. *C*. *jejuni* stored in dark, oxygenated conditions grew significantly better than those stored in a light, oxygenated environment, indicating that the toxic effects of oxygen may be photochemically induced [27,29]. Further investigation of this trait could lead to light-based interventions during meat processing. Cell density also affects aerotolerance, and high cell densities of *C*. *jejuni* grew better in aerobic conditions than low densities [12]. Cells in the exponential growth phase survived oxidative stress better [79]. Exposure to acid stress increased *katA* expression in *C*. *jejuni*, and tolerance to H_2_O_2_ increased after acid stress [80]. These studies clearly illustrate that multiple confounding variables may exist when testing the aerotolerance of a particular strain and emphasize the importance of considering environmental factors when evaluating aerotolerance in vitro. These factors likely impact aerotolerance in *Campylobacter* during meat processing.

### 3.4. Influence of Intrinsic Factors on Aerotolerance

*Campylobacter* also employs several intrinsic strategies to enhance its survival during oxygen exposure. While a more detailed discussion of genetic mechanisms is provided later in this review, here we highlight studies that observed adaptations to aerobic conditions without in-depth or direct study of the genes involved. The viable-but-nonculturable (VBNC) state may be one strategy used by *Campylobacter* to survive in O_2_. A large increase in coccoid VBNC cells was observed in aerobic conditions and during exposure to oxidative stress [54,55,56]. The contribution of individual strains in the adaptation of *Campylobacter* to aerobic growth should also be considered. Subcultures taken from *C*. *jejuni* strains cultured in aerobic conditions on blood agar survived and grew in aerobic environments, suggesting an adaptation to aerobic conditions [81]. Cell morphology remained unchanged, and *C*. *jejuni* retained its ability to colonize mice, which eliminates the possibility that the cells were simply entering the VBNC state [81]. Furthermore, strains that adapted to aerobic conditions showed better survival at low temperatures, indicating that adaptation to aerobic conditions may affect thermotolerance in *C*. *jejuni* [81]. One study confirmed that selected strains of *C*. *jejuni* could be subcultured to induce aerobic growth using nutrient agar [82], and another study showed that waterborne isolates of *C*. *jejuni* were able to adapt to aerobic conditions via repeated subculturing [83]. In a recent study, approximately 52% (43/83) of *C*. *jejuni* strains acclimated to aerobic conditions after repeated subculturing [84], suggesting adaptation to oxygen may be strain-specific. Furthermore, aerotolerance in *C*. *coli* increased via serial passage in aerobic conditions [85]. Whole genome sequencing of the adapted *C*. *coli* strain revealed 23 point mutations that were absent in the parental strain, and none of these were in genes involved in the oxidative stress response [85]. This study demonstrates that an underlying genetic change is present in the development of aerotolerance and involves additional genes not known to be involved in the oxidative stress response. Furthermore, this result suggests that aerotolerance can change in response to prevailing environmental conditions. Additional studies are needed to identify the genetic changes underway in the adaptation to aerobic conditions and their potential role in meat processing and other environments. We can speculate that adaptation to oxygen exposure is likely occurring during meat processing, but further research is necessary to confirm this hypothesis and identify the conditions under which adaptation is most likely to occur. Table 1 summarizes the numerous studies documenting the effect of growth conditions on *Campylobacter* aerotolerance. It is important to note that results can vary between studies, and researchers using different growth conditions may have disparate results, even when factors such as the strain used are consistent. For consistency, it would be helpful to adopt a standardized testing method for aerotolerance.

## 4. Correlations between Aerotolerance and Virulence or Survival Ability

Positive correlations have been observed between the aerotolerance of specific *Campylobacter* strains and their level of virulence and antibiotic resistance. One recent study examined the prevalence of eight virulence genes in 70 *C*. *jejuni* strains and found that virulence genes were more prevalent in hyper-aerotolerant strains of *C*. *jejuni* than in aerosensitive strains [78]. Certain virulence genes, including *cadF*, *iam*, *pldA*, *docA*, *peb1*, and *flaA*, were detected more frequently in aerotolerant and hyper-aerotolerant *C*. *jejuni* strains isolated from duck meat than in aerosensitive strains [15]. However, virulence gene frequency was independent of aerotolerance in strains isolated from chicken [15], so the previous correlation may not be consistent across different types of meat. The underlying reason for this discrepancy is unclear due to the limited number of available studies, and further research is needed to validate these findings and determine the contributing factors. Expression of three virulence genes in *C*. *jejuni* was reported to be similar in both aerotolerant and aerosensitive strains [86]; however, it is noteworthy that aerotolerant strains in this study were defined as surviving for at least 120 h with a reduction in CFU/mL less than log_2_ [86]. Overall, the correlation between aerotolerance and virulence is inconclusive due to the limited number of studies available and warrants further study.

Antibiotic resistance has a potential impact on the survival of *Campylobacter* in oxygenated environments since resistance to metronidazole and fluoroquinolone correlated with aerotolerance [12,87]. Another report describes *cmeG*, a putative efflux transporter in *C*. *jejuni*, as having a potential role in antibiotic resistance and oxidative stress, which could also impact aerotolerance [88].

Aerotolerance in *Campylobacter* also correlates with the ability to survive other stressful conditions. For example, a high percentage of cold-tolerant *C*. *jejuni* strains were also aerotolerant and showed higher survival in response to refrigeration and freeze-thaw stress [16,19,78,89,90]. However, there was no correlation between aerotolerance in different *Campylobacter* strains and their ability to survive in refrigerated milk [91]. Interestingly, one study found that *Campylobacter* survived refrigeration better when kept under aerobic rather than microaerobic conditions, perhaps suggesting a link between *Campylobacter*’s cold and oxidative stress responses [89]. A genetic link between the cold stress response and oxidative stress response has also been suggested [92,93,94], which is discussed further in Section 5.2 of this review. Strains of *C*. *jejuni* with increased aerotolerance survived better than aerosensitive strains when exposed to peracetic acid [90]. It is important to mention that evidence is conflicting as to whether aerotolerance correlates with resistance to heat and osmotic stress [19,90]. Currently, the number of published studies examining the correlation between aerotolerance and other environmental traits is relatively small, and further research is needed to explore the significance and prevalence of these correlations.

## 5. Mechanisms of Aerotolerance

### 5.1. Role of Oxidative Stress Genes katA, sodB, and ahpC on Aerotolerance

Several studies have addressed the mechanisms utilized by *Campylobacter* spp. for surviving aerobic conditions, and these help determine appropriate strategies for preventing aerotolerant strains from surviving in and contaminating our food supply. Here, we highlight three well-studied oxidative stress genes: *katA*, *sodB*, and *ahpC*. The gene *katA* encodes a catalase that prevents the accumulation of H_2_O_2_ and reduces oxidative stress in *Campylobacter* [52]. Consequently, *katA* mutants of *Campylobacter* are more sensitive to H_2_O_2_ and aerobic conditions [80,95,96]. Additionally, aerotolerant *C*. *jejuni* strains have higher levels of catalase activity and/or increased expression of *katA* [54,90,97,98,99].

SOD, which is encoded by *sodB* in *Campylobacter*, protects cells against reactive oxygen species (ROS) [96,99,100]. Aerotolerant strains of *C*. *jejuni* usually exhibit high levels of SOD activity and upregulated expression of *sodB* [54,90,98,101], whereas *sodB* mutants are more susceptible to oxidative stress [96,102,103]. Interestingly, one study reported that *sodB* expression in *C*. *jejuni* did not increase in response to oxidative stress; however, the same researchers showed that a *sodB* mutant had impaired oxidative stress tolerance [103]. These results suggest that a stable level of *sodB* expression is required for mounting a response to ROS [103]. 

The peroxide reductase encoded by *ahpC* is involved in mitigating damage due to oxidative stress in *Salmonella typhimurium* and *Escherichia coli* [104]. In *Campylobacter*, *ahpC* expression increased in aerobic conditions [96,98,105], and mutating *ahpC* in a hyper-aerotolerant strain impaired aerotolerance [17]. In response to atmospheric O_2_ and cumene hydroperoxide, *ahpC* mutants exhibited decreased survival rates; however, a *C*. *coli ahpC* mutant exhibited increased tolerance to H_2_O_2_ [106], indicating that *ahpC* does not improve resistance in all *Campylobacter* spp.

The *katA*, *sodB*, and *ahpC* genes have been intensively studied for their role in oxidative stress and *Campylobacter* aerotolerance (Table 2). One study hypothesized that these genes could be used to develop a live, attenuated vaccine for chickens [107]. Furthermore, chickens pre-infected with *ahpC* or *katA* mutants prior to colonization with a wild-type strain showed reduced numbers of *Campylobacter* in their feces as compared to those colonized by the wild-type only [107]. However, pre-colonization with a *sodB* mutant did not reduce the number of *Campylobacter* cells in feces [107]. Further study of these and other genes could be useful in the development of a poultry vaccine for *Campylobacter*.

### 5.2. Role of Temperature on Aerotolerance

Along with the observation that temperature can affect aerotolerance, it has been reported that multiple genes involved in the heat stress response of *Campylobacter* also influence aerotolerance. HtrA is a widely-conserved serine protease that has a role in the heat shock response and in the degradation of heat-denatured or misfolded proteins [133,134]. Two studies reported that aerotolerance at 17–18% O_2_ was reduced in a *C*. *jejuni htrA* mutant [119,120]; however, the mutant was not vulnerable to cumene hydroperoxide or paraquat, which are known to increase the accumulation of ROS [119]. One study found evidence that the temperature sensitivity of an *htrA* mutant might be dependent on the level of oxidative stress affecting the mutant, suggesting that bacterial sensitivity to heat may be intertwined with its sensitivity to oxygen [135]. The transcriptional repressor encoded by *hspR* is involved in the response to temperature stress, and *hspR* mutants of *C*. *jejuni* exhibited lower aerotolerance as compared to wild-type strains [121]. Another study reported a novel protein designated Cj62 that showed increased accumulation in *C*. *jejuni* during both aerobic conditions and heat stress [122]. Another important related stress response to discuss is that of cold stress. A few studies have suggested some mechanistic relationship between cold stress and aerotolerance. For example, certain genes known to play a role in resistance to oxidative stress, in particular, *sodB*, may also contribute to the ability of *C*. *jejuni* to resist cold stress and freeze-thaw conditions [92,93,94]. It was also reported that *sodB* mutants of *C*. *coli* are more vulnerable to cold stress than the wild-type but only in the presence of oxygen [92], and this was later demonstrated in *C*. *jejuni*, as well [94]. A protein encoding for a putative periplasmic cytochrome C peroxidase, Cj0358, was also shown to have increased activity under cold shock [93]. As noted earlier in Section 4 above, *Campylobacter* shows enhanced survival during refrigeration under aerobic rather than microaerobic conditions, suggesting a close link between the two stress responses [89]. The connection between certain oxidative stress genes, such as *sodB*, and the cold stress response could explain this phenomenon.

### 5.3. Role of perR, mutY, and cosR on Aerotolerance

It is likely that transcriptional regulators may play a role in aerotolerance. The mutation of *perR*, *mutY*, and *cosR* increased aerotolerance in *C*. *jejuni* [106,108,109,124,125,131], which suggests a regulatory role for these genes. Inactivation of the *perR* repressor in *C*. *jejuni* increased aerotolerance and resistance to H_2_O_2_ and resulted in increased expression of *katA*, *ahpC*, *rrc*, and *trxB* [109]. This confirmed previous findings where a *perR* mutant displayed increased expression of *ahpC* and *katA* [108]. Transcriptomic analysis of a *C*. *jejuni perR* mutant as compared to the wild-type indicated that *perR* is involved in the regulation of many genes, including several related to the oxidative stress response [124]. It is helpful to note that *perR* transcription was reduced in the presence of iron, and *perR* expression may be influenced by the *fur* gene, which is iron-regulated [46,47,48]. This interaction could establish a link between the role of iron in aerotolerance and the *perR* repressor. A mutation in the DNA repair enzyme MutY in *C*. *jejuni* resulted in enhanced resistance to oxidative stress [131]; this led investigators to propose that the *mutY* mutant resulted in *perR* mutations, leading to derepression of various oxidative stress genes controlled by PerR [131]. The response regulator CosR controls multiple proteins involved in the oxidative stress response of *C*. *jejuni*, including AhpC, SodB, Dps, Rrc, and LuxS; CosR acts as a repressor for all these proteins except AhpC [125]. These interactions were explored by knocking down the expression of *cosR* in *C*. *jejuni*, which resulted in a strain that was more resistant to oxidative stress [125]. Transcriptional regulators are likely to have a significant effect on aerotolerance due to their ability to influence the expression of multiple genes.

### 5.4. Role of Biofilms and Polysaccharides on Aerotolerance

As discussed previously, biofilm formation is likely to play a crucial role in enhancing aerotolerance. Select genes involved in biofilm formation and quorum-sensing influence aerotolerance [43,123,129]. CsrA has a role in biofilm formation and the invasion of host cells, and a *csrA* mutant of *C*. *jejuni* displayed reduced resistance to oxidative stress [123]. The *peb4* gene is presumably involved in biofilm formation [43,136], and a *peb4* mutant was less culturable in aerobic conditions than the wild-type *C*. *jejuni* [43], which could explain why the mutant did not grow well in aerobic conditions. The *luxS* gene is involved in the biosynthesis of autoinducer-2, a quorum-sensing molecule in *C*. *jejuni*, and a *luxS* mutant was more impaired by exposure to H_2_O_2_ and cumene hydroperoxide than the wild-type [129]. Biofilms remain a good target for control measures to reduce *Campylobacter* contamination in our food supply.

Capsule formation is another strategy employed by aerotolerant strains of *C*. *jejuni* to survive aerobic conditions, and a recent study demonstrated that a layer of surface polysaccharides is formed when *C*. *jejuni* is exposed to aerobic conditions [127]. Moreover, a mutant defective in both the *kpsS* and *waaF* genes, which are involved in the synthesis of capsular polysaccharides and lipooligosaccharides (LOS), respectively, was impaired in aerotolerance [127]. Another noteworthy gene is *htrB*, which is involved in the synthesis of lipid A, another component of LOS [137]. Expression of *htrB* was higher in aerotolerant strains of *C*. *jejuni* than aerosensitive strains, although it should be noted that the study defined aerotolerant as survival for more than 120 h under aerobic conditions [86]. Targeting genes involved in capsule formation and membrane integrity may be a good strategy for reducing aerotolerance.

### 5.5. Role of Genes Involved in Iron Metabolism and Other Metals

Certain genes involved in the metabolism of iron and other metals appear to contribute to aerotolerance. One gene that functions in the oxidative stress response is *fdxA*, which encodes a putative ferredoxin; *fdxA* mutants of *C*. *jejuni* were shown to have reduced aerotolerance capabilities, and the FdxA protein may have a role in the reduction of AhpC [114,115]. Furthermore, a *C*. *jejuni* mutant defective in the ferritin gene *cft* showed more sensitivity to H_2_O_2_ and paraquat than the wild-type [118], and *cft* was overexpressed when bacteria were exposed to paraquat [103]. Another study demonstrated that a mutation in *czcD*, which encodes a zinc exporter, decreased the tolerance of *Campylobacter* to H_2_O_2_ [106]. A *C*. *jejuni* strain containing a mutation in *herA*, which encodes a hemerythrin, displayed a reduced growth rate in aerobic conditions and may have a role in protecting Fe-S cluster proteins from peroxide damage [113]. In *C*. *jejuni*, the Dps protein protected bacterial cells from H_2_O_2_ exposure by binding to and sequestering iron [117], and *dps* expression was upregulated in aerobic conditions [98]. Inactivation of *tonB2*, which presumably encodes a protein involved in iron acquisition, increased sensitivity to H_2_O_2_ [111]. 

### 5.6. Role of Other Genes on Campylobacter Aerotolerance

Numerous other genes have been identified with potential roles in the oxidative stress response of *Campylobacter* or in the development of aerotolerance. For example, *rrpB* (also known as *cj1556*) functions as a transcriptional regulator that modulates genes involved in the oxidative stress response, and a *C*. *jejuni rrpB* mutant exhibited reduced aerotolerance [126,138]. A mutation in *rrpA* (previously *cj1546*) rendered *C*. *jejuni* more sensitive to H_2_O_2_ and aerobic conditions [126]. The *fldA* gene, which encodes a flavodoxin with potential involvement in oxidative stress, was overexpressed when *C*. *jejuni* was exposed to paraquat [103]. In another study, the Type VI Secretion System (T6SS) was shown to have a role in oxidative stress since a mutation in the T6SS gene *tssD* increased sensitivity to ROS [128].

Mutations in the *tpx* and *bcp* genes, which encode peroxidases in *C*. *jejuni*, resulted in normal growth under microaerobic conditions but decreased growth under aerobic conditions as compared to the wild-type [110]. It is noteworthy that *tpx* was upregulated in aerobic conditions [98], and the *tpx/bcp* double mutant was more affected than strains containing a single mutation [110]. In *C*. *jejuni*, *msrA* and *msrB* are involved in the oxidative stress response, and mutants defective in these genes exhibited decreased tolerance to compounds that induce oxidative stress [116]. Mutation of *pstC*, a gene with a putative role in phosphate transport, also resulted in increased sensitivity of *C*. *jejuni* to H_2_O_2_ [111].

*C*. *jejuni* mutants with a defective *cmeG* gene exhibited increased susceptibility to H_2_O_2_ and several antibiotics [88]. Furthermore, *C*. *jejuni* mutants disrupted in selected motility genes, including *flgP*, *flgI*, *flgK*, *flgL*, *flgR*, *flhB*, *flgD*, *flgH*, and *pseB*, displayed increased sensitivity to oxidants, as did a *motA*/*motB* double mutant [111]. It seems plausible that motility is important for oxidative stress tolerance since mutations in the flagellar apparatus that impaired motility also increased sensitivity to oxidative stress, whereas mutations to the flagellum that did not affect motility showed no increase in sensitivity [111]. This research suggests that targeting the bacteria’s motility may be a viable strategy for inhibiting its aerotolerance.

### 5.7. Proteins Known to Be Involved in Campylobacter Aerotolerance

At the protein level, increased activity of NADH oxidase was observed in an aerotolerant variant of *C*. *jejuni* [54]. Two enzymes involved in redox reactions, TrxB (thioredoxin reductase) and OorA (2-oxoglutarate:acceptor oxidoreductase), were overproduced when *C*. *jejuni* was exposed to aerobic conditions [98]. Furthermore, an aerotolerant strain of *C*. *jejuni* designated Bf contained a mutated version of *oorD* [130,139]. Several proteins that function in the TCA cycle were more abundant in the Bf strain in aerobic conditions as compared to a microaerobic environment [98]. In *C*. *jejuni*, a PerR-regulated protein designated Rrc, which is similar to the rubredoxin oxidoreductases and rubrerythrins, was shown to have a role in the oxidative stress response [109,111,112]. AcnB, a protein involved in the post-transcriptional regulation of oxidative stress proteins in *E*. *coli* [140], may have a similar role in *C*. *jejuni*, but further study is needed [111]. Stress response protein FusA and structural support protein MreB, along with virulence factors CadF and FlaA, were overproduced in *C*. *jejuni* in response to paraquat [103].

### 5.8. Genes and Proteins Involved in Aerotolerance That Require Further Characterization

Several unidentified genes have also been implicated in aerotolerance. One study reported a gene encoding a catalase-like heme-binding protein that was present in both aerotolerant and aerosensitive strains of *C*. *coli*; interestingly, this gene was not identified in *C*. *jejuni* [21]. In an aerosensitive strain of *C*. *coli*, the gene encoding the catalase-like protein was expressed at twofold higher levels when grown in aerobic conditions than when cultured in microaerobic conditions [21]. Another study used transcriptomics to identify multiple genes (e.g., *cj0203*, *cj0264c*, *cj0415*, *cj0425*, *cj0628*, *cj0629*, and *cj0864*) that may contribute to aerotolerance of *C*. jejuni [12]. In another report, a *C*. *jejuni* mutant disrupted in *cj1386* was less resistant to H_2_O_2_ and had lower levels of catalase activity as compared to the wild-type [132]. The researchers suggested that Cj1386 may have a role in the trafficking of heme to KatA [132]. Several uncharacterized proteins, namely Cj1371, Cj1476c, and Cj0012c, were overproduced during oxidative stress [103]. In another report, increased sensitivity to oxidative stress was observed in *C*. *jejuni* with mutations in *cj0062c*, *cj0344*, *cj0947c*, and *cj1388* [111].

Identification of the mechanisms and genes involved in aerotolerance is important for understanding how aerotolerance develops and how it might be prevented or attenuated. Various proteins highlighted in these studies may be good targets for the development of inhibitors. Targeting these proteins with specific inhibitors could reduce *Campylobacter*’s ability to withstand the stresses encountered during food processing and make the bacteria more susceptible to existing food safety interventions. Although many oxidative stress response genes have been identified in *Campylobacter*, further studies are needed to clarify how these genes function and interact.

## 6. Concluding Remarks

**Figure 1:**
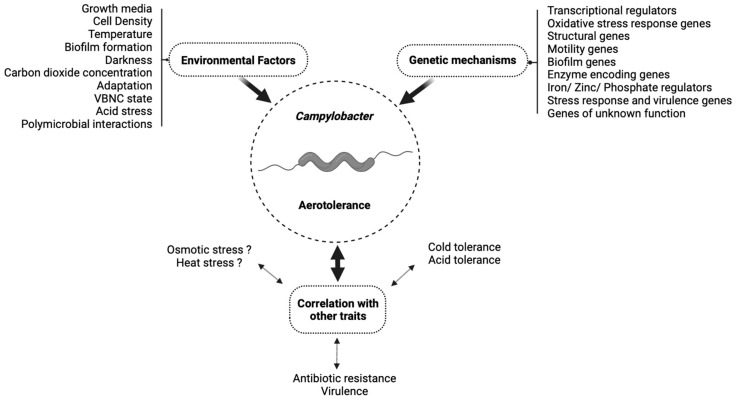
A schematic diagram summarizing mechanisms of *Campylobacter* aerotolerance and its interactions with environmental factors and traits discussed in this review.

The remarkable ability of *Campylobacter* spp. to survive oxygen exposure long enough to contaminate our food supply despite its microaerophilic nature remains a mystery. However, recent studies involving the prevalence of aerotolerant *Campylobacter* strains suggest that aerotolerance could be the key to resolving this question. Aerotolerance is strongly influenced by multiple environmental factors, including the growth media, temperature conditions, and polymicrobial interactions. The brand and age of media must also be carefully considered for its potential impact on aerotolerance, underscoring the importance of consistent media formulations for aerotolerance testing. Biofilm formation, especially polymicrobial biofilms, is extremely beneficial to aerotolerance and could be a major contributor to survival. Additionally, conditions of refrigeration and darkness appear to enhance aerotolerance in *Campylobacter*. It appears that a close tie exists between *Campylobacter*’s responses to cold stress and oxidative stress, both in terms of genetics and mutual reinforcement. The VBNC state appears to be an important factor in survival under aerobic conditions. Multiple studies have also revealed that *Campylobacter* can be subcultured under aerobic conditions to increase aerotolerance, which could explain how aerotolerance develops. Unfortunately, very few genes have been well-studied for their roles in aerotolerance, and many remain unidentified. Further research is warranted to further clarify the role of environmental factors in aerotolerance and the genetic mechanisms that control it. Additionally, although many avenues for food safety interventions have been outlined by the studies discussed, research into the clinical implications of aerotolerance is severely lacking. Only one study on the prevalence of aerotolerance in clinical strains was identified [16], and no studies were found to examine the potential impact of aerotolerance on clinical outcomes. As shown in Figure 1, there are several genetic mechanisms that are believed to be involved in *Campylobacter* aerotolerance and there are numerous environmental factors that were shown to influence this trait. It is also clear that there is a possible correlation between *Campylobacter* aerotolerance and some other traits, such as virulence or antibiotic resistance, that needs to be confirmed with more research.

## 7. Future Directions

Many aspects of aerotolerance in *Campylobacter* spp. require further investigation. First, it would be helpful to increase aerotolerance testing for clinical isolates to evaluate whether aerotolerant strains are more likely to survive long enough to infect humans. This would help us further assess the relevance of aerotolerance to infections in humans. Similarly, further study of possible correlations between aerotolerance and virulence or antibiotic resistance would be extremely valuable in monitoring and mitigating the spread of aerotolerant *Campylobacter* strains. Additionally, research into the clinical outcomes of infection with aerotolerant strains is important for understanding the potential for complications and long-term effects such as Guillain-Barré syndrome. It would also be valuable to assess the prevalence of aerotolerance in isolates obtained from environmental sources such as water since this will help us understand the dispersal and spread of aerotolerant strains. To our knowledge, studies on the prevalence of aerotolerance in environmental strains of *Campylobacter* have not been conducted. Further studies are also needed to address how strains become aerotolerant when subjected to repeated subculturing and whether the conditions encountered during meat processing are encouraging this change. To understand the underlying mechanisms of aerotolerance, additional genomic studies are needed. Numerous genes require additional characterization to confirm their function in aerotolerance, and many unknown genes need to be further investigated to clarify their role in aerotolerance. Since many genes in the *Campylobacter* genome remain unknown or hypothetical, there is plenty of opportunity for further study. Finally, it is vital that a standardized method of testing for aerotolerance is adopted in the scientific community to keep data consistent and allow for more accurate comparisons between studies since aerotolerance is greatly influenced by environmental conditions.

## Figures and Tables

**Table 1:** Environmental conditions involved in aerotolerance.

Environmental Condition	References
**Growth Media and Supplements**	
Iron, iron sources, norepinephrine	[25,33,36]
Blood	[30,34,36,56]
Iron sulfate, sodium bisulfite, and sodium pyruvate (FBP)	[26,27,30,34,38,56]
Charcoal, iron sulfate, sodium pyruvate	[36]
C4-dicarboxylates, C3-monocarboxylates, and sodium bicarbonate	[40,53]
Pyruvate	[32,52]
Hemin	[34]
Bisulfite	[28,31,32]
Different brands of complex media	[32]
Chicken or beef liver juice, meat juice	[39]
Beef extract	[40]
Formate ‡	[71]
Serine	[57]
Dithionite or histidine	[29]
Allopurionol, azelaic acid, caffeine, cimetidine, TEMPOL	[32]
**Enzymes**	
Superoxide dismutase	[27], [29] *, [30], [32] *, [58]
Catalase	[27,29]
Oxyrase	[34,50]
**Polymicrobial Interactions**	
Co-cultivation with *Pseudomonas* spp.	[58,62]
Co-cultivation with *S. aureus*	[60]
Co-cultivation with *B. subtilis*	[61]
Co-cultivation with *Salmonella*	[59]
Membrane fragments from *P. aeruginosa* and *E. coli*	[62]
Co-cultivation with amoeba	[63,64]
Bacteriophage exposure	[65]
**Other Environmental Conditions**	
Biofilm formation	[19,42,43,44,45,55,67,68,69,71,72]
Darkness	[27,29]
Temperature	[11,28,31,77]
Cell density	[12]
Subculturing and air-adaptation	[81,82,83,84,85]
VBNC state	[54,55,56]
>97% carbon dioxide ‡	[78]
Acid stress	[80]

* No benefit to aerotolerance; ‡ Inhibits aerotolerance.

**Table 2:** Genes and Proteins Involved in the Mechanisms of Aerotolerance.

Genes/Proteins Involved in Aerotolerance	References
**Oxidative Stress Response**	
*katA*	[54,80,90,95,96,97,98,103,105,107,108,109]
*ahpC*	[17,96,98,105,106,107,108,109]
*sodB*/SOD	[54,90,96,98,100,101,102,103,107]
*tpx*	[98,110]
*bcp*	[110]
*rrc*	[109,111,112]
*trxB*	[98,109]
*herA*	[113]
*fdxA*	[114,115]
FldA	[103]
*msrA* and *msrB*	[116]
**Iron, Zinc, or Phosphate Regulation**	
*dps*	[98,117]
*cft*	[103,118]
*tonB2*	[111]
*czcD*	[106]
*pstC*	[111]
**Stress Responses and Virulence**	
*htrA*	[119,120]
HspR	[121]
Cj62	[122]
FusA, CadF, FlaA	[103]
*cmeG*	[88]
**Transcriptional Regulators**	
*csrA*	[123]
*perR* †	[106,108,109,124]
*cosR* †	[125]
*rrpA* and *rrpB/cj1556*	[126]
*acnB*	[111]
**Structural Components**	
*kpsS* and *waaF/rfaF*	[127]
*htrB*	[86]
*tssD*	[128]
MreB	[103]
**Biofilm Formation and Motility**	
*peb4*	[43]
*flgP*, *flgI*, *flgK*, *flgL*, *flgR*, *flhB*, *flgD*, *flgH*, *pseB*, *motA*, *motB*	[111]
**Other Enzymes**	
*luxS*	[125,129]
*oorA*	[98]
*oorD*	[130]
MutY †	[131]
**Unknown Functions**	
*cj0203*, *cj0264c*, *cj0415*, *cj0425*, *cj0629*, *cj0864*	[12]
*cj1386*	[132]
Unidentified catalase-like heme-binding protein	[21]
*cj1371*, *cj1476c*, *cj0012c*	[103]
*cj0062c*, *cj0344*, *cj0947c*, *cj1388*	[111]

† Mutating these genes appears to result in an increase in aerotolerance rather than a decrease.
